# Dermatology

**Published:** 1910-09

**Authors:** J. A Nixon


### DERMATOLOGY.

**Organotherapy in sclerodermia** forms the subject of a comprehensive paper by Roques,[1] in which he has collected a large number of published cases treated by the administration of animal organs or their extracts. He premises that the multiplicity of remedies in vogue for sclerodermia is due to their inefficacy, but organotherapy appears to mark an advance provided the recent theories are correct that the disease depends on alterations of the vascular glands (pituitary, suprarenal and especially thyroid).

**Thyroid** treatment is based on the frequency with which abnormalities of the thyroid gland—hypertrophy, atrophy, Graves's disease or myxœdema—have been observed to co-exist with sclerodermia.

Roques gives a tabular summary of sixty-seven cases of diffuse sclerodermia (in some of which the thyroid is stated to have been altered) where thyroid extract was administered with the following results :—

| | | |
|---|---|---|
| Cases cured .. .. .. | 4 or | 5.97 per cent. |
| ,, improved .. .. | 32 or | 47.76 per cent. |
| ,, not improved .. | 31 or | 46.18 per cent. |

Percentage of favourable results, 67.73.

A few instances of circumscribed sclerodermia treated with thyroid extract have been collected : out of ten cases one was cured and six improved. The relation between **suprarenal** disease and sclerodermia is based on very slender evidence, but at three necropsies the suprarenals have been described as diseased. Three cases are quoted with definite improvement after administration of suprarenal extracts, while in a fourth case under Roques's observation there was slight amelioration.

The **pituitary body** has been accused of being at fault in a few cases of sclerodermia, but therapeutically it has proved inert.

Defects of the **genital organs** or functions associated with sclerodermia have led to the administration of testicular extract in two cases without benefit, and ovarian extract has proved equally useless. Mesenteric gland extracts (based on a theory of gastro-intestinal auto-toxæmia) has been administered by Schwerdt, and his results (quoted by Roques) were distinctly favourable in five cases.

Finally, **polyglandular combinations** have been employed in treatment. Renon has seen a case of sclerodermia lose its induration after several weeks of thyro-ovarian treatment. On the other hand, combined pituitary and thyroid extract yielded no favourable results to different observers. On the whole Roques is of the opinion that organotherapy has given more encouraging results than other remedies, and that not only

[1] *Ann. de Derm. et Syph.*, 1910, vii, 383.

thyroid but a trial of other glandular extracts separately or combined should be essayed, bearing in mind that it is only in the early stages that a cure can be reasonably expected, "atrophy of the skin cannot yield to any remedy."

**The congestive action of potassium iodide on tuberculosis of the skin** is discussed by Audry.[1] This congestive action has long been recognised in pulmonary tuberculosis. Audry has observed that the administration of four to six grammes of potassium iodide daily is followed after two or four days by very definite local congestive phenomena in lupus vulgaris, tuberculous ulceration, Darier's keratosis follicularis and lupus erythematosis. The congestion disappears on discontinuing the drug. The author has not found that this action exercises any ill effects on the disease, but rather the reverse. He is content, however, to record the fact without expressing a definite opinion as to its value. He admits that potassium iodide plays the part of accelerator in a number of epithelial neoplasms, and is manifestly harmful in leprosy, but in these cases it does not produce the rapid congestion seen in tuberculides. The reaction in tuberculosis is much more transient, slight and inconstant than that provoked at times round syphilides at the outset of mercurial treatment. In some subjects it may almost be compared to a tubercular reaction of moderate intensity.

**Experimental production of pigment in vitiligo.** Audry[2] summarises the observations of Stein[3] thus, "The pigment can be restored in vitiligo either by chemical radiations (Buschke's researches with the Kromayer lamp) or with thermal agents (carbon dioxide snow). The pigment thus formed appears in disseminated spots after a variable incubation period. It is intra- and extra-cellular, and resembles the normal pigment. It does not give any iron reaction, it is acid, soluble in hydrogen peroxide, is stained green by polychrome blue, and responds to the reaction for melanin."

J. A. NIXON.

### Reviews of Books.

**Severest Anæmias: their Infective Nature, Diagnosis and Treatment.** By WILLIAM HUNTER, M.D. Vol. I. Pp. xv, 226. London: Macmillan & Co. 1909.

Hæmatologists have evolved a remarkable terminology for their particular branch of medical science; it tends to confuse the average reader. Whether the bold stand taken by the writer

[1] *Ibid.*, p. 405. [2] *Ibid.*, p. 415.

[3] *Arch. f. Derm. u. Syph.*, 1909, xcvii, 163.

